# Mysterious Mechanisms of Memory Formation: Are the Answers Hidden in Synapses?

**DOI:** 10.7759/cureus.5795

**Published:** 2019-09-28

**Authors:** Viraj V Joshi, Nishita D Patel, Muhammad Awais Rehan, Annapurna Kuppa

**Affiliations:** 1 Neuropsychiatry, California Instititute of Behavioral Neurosciences and Psychology, Fairfield, USA; 2 Research, California Institute of Behavioral Neurosciences & Psychology, Fairfield, USA; 3 Miscellenous, California Institute of Behavioral Neurosciences & Psychology, Fairfield, USA; 4 Internal Medicine and Gastroenterology, University of Michigan, Ann Arbor, USA

**Keywords:** synaptic function of neurons and memory formation, hippocampus and memory formation, synaptic plasticity and long term potentiation, alzheimer disease and synaptic dysfunction, engram cells and memory

## Abstract

After decades of research on memory formation and retention, we are still searching for the definite concept and process behind neuroplasticity. This review article will address the relationship between synapses, memory formation, and memory retention and their genetic correlations. In the last six decades, there have been enormous improvements in the neurochemistry domain, especially in the area of neural plasticity. In the central nervous system, the complexity of the synapses between neurons allows communication among them. It is believed that each time certain types of sensory signals pass through sequences of synapses, these synapses can transmit the same signals more efficiently the following time. The concept of Hebb synapse has provided revolutionary thinking about the nature of neural mechanisms of learning and memory formation. To improve the local circuitry for memory formation and behavioral change and stabilization in the mammalian central nervous system, long-term potentiation and long-term depression are the crucial components of Hebbian plasticity. In this review, we will be discussing the role of glutamatergic synapses, engram cells, cytokines, neuropeptides, neurosteroids and many aspects, covering the synaptic basis of memory. Lastly, we have tried to cover the etiology of neurodegenerative disorders due to synaptic dysfunction. To enhance pharmacological interventions for neurodegenerative diseases, we need more research in this direction. With the help of technology, and a better understanding of the disease etiology, not only can we identify the missing pieces of synaptic functions, but we might also cure or even prevent serious neurodegenerative diseases like Alzheimer’s disease (AD).

## Introduction and background

Donald Hebb, the “father of neuropsychology”, proposed in his book, “The Organization of Behavior” (1949), that memories are stored in the mammalian brain as synaptic connections between neurons for the activity of learning and memory formation. Hebb postulated that “increments in synaptic efficacy occur during learning when the firing of one neuron repeatedly produces firing in another neuron to which it is connected.” We refer to a synapse that is modified in this manner as a “Hebb synapse”. The Hebb synapse is the theoretical foundation for many neurobiological and computational models of synaptic plasticity; it has also revolutionized thinking about the nature of the neural mechanisms of learning and memory formation [1].

Synapses are the junction from one neuron to the next, which together regulate the direction in which nerve signals pass through the nervous system. On many occasions, our brains can make us feel like we have experienced the same situation in the past, although in reality, we have not. This phenomenon is called “déjà vu”. How does this happen? Every time particular signals pass through specific sequences of synapses, these synaptic sequences become accustomed to the same type of stimulus. These sequences are so complicated that sometimes they overlap with each other and give us the perception of experiencing a known event even though it may be the first time we are experiencing it [2]. Memory is a modification of certain temporal essences of past incidents, such as the repetition of a stimulus or the coincidence of multiple stimuli. In simple language, neurons are molecular and cellular windows, which collectively store data from the past. For detection and response to a particular situation, the nervous system uses its capacity to encode and store memory at the molecular, cellular, synaptic, and circuit levels [3]. Most storage occurs in the cerebral cortex; however, a small amount of information can be stored even in the basal regions of the brain and the spinal cord [2]. The hippocampus spans the posterior-anterior extent of the base of the temporal lobes and plays a crucial role in learning, memory, and recognition of innovation. The strengthening and weakening of synaptic connections between neurons are called long-term potentiation (LTP) and long-term depression (LTD), respectively; these form the basis for normal cognitive function [4].

Long-lasting activity-dependent alterations of the synaptic structure and their transmission efficiency is called synaptic plasticity [5]. Research on synaptic plasticity opens opportunities in understanding molecular mechanisms of memory formation. Several theories have been proposed to explain the relationship between synapses and memory formation. In this review, we will be discussing the role of glutamatergic synapses, engram cells, cytokines, neuropeptides, neurosteroids, and many more mechanisms that form the synaptic basis of memory formation. One of the most popular theories is the involvement of glutamatergic and gamma-aminobutyric acid (GABA) synapses, especially postsynaptic glutamate receptor density [6]. It is believed that memories residing in engram cells are distributed across the brain, but mainly they are the subset of the basolateral amygdala (BLA) [3]. However, it is generally accepted that synapses encode memories, and that the site-specific substrate within these engram cells remains theoretical [7]. In this review, we address some of the important evidence that has shown the Aβ oligomer-centric hypothesis as well as some of the key findings concerning the impact of Aβ oligomers on synapses at a morphological and functional level [8]. We have also reviewed evidence on the molecular events that occur during the formation and stabilization of dendritic spines, and the signaling pathways regulating these processes, to understand the mechanisms of learning and memory [9]. Higher order cognitive functions, such as decision making and spatial and language learning, were also discovered from neural plasticity mechanisms [10].

This review also covers how knowledge of cellular and molecular mechanisms of synaptic plasticity can provide a better understanding of learning and memory formation. Understanding how Aβ oligomers target synapses provides an important framework for ongoing AD research, which can lead to the evolution of successful therapeutic strategies designed to alter or perhaps reverse the course of the disease.

## Review

Methods

Inclusion Criteria, Search Strategy, and Data Extraction

We have identified primary studies on the basis of the question, “What is the relation between synapses and memory? What is the correlation between the disease process and synaptic dysfunction?”

To accomplish our goal, a search strategy was developed, in which we included the abstracts of review articles of human studies, clinical trials, bibliographies, and books from databases like PMC, PubMed, Elsevier, and Wiley Online Library. All relevant and potentially eligible studies published in peer-reviewed journals were considered. The articles from various databases were dated between 1 January, 2013 and 22 June, 2018. However, one of the extraordinary quotes written by the father of neuroscience, Dr. Hebb, was taken from an article written in a 1998 journal. Duplicate studies were removed not by digital object identification, but by manual comparison of titles, authors, publication dates, and article metadata. The last search was carried out on 20 July, 2018.

For each study, only the abstracts were used due to strict inclusion criteria of publication date, the enormous amount of data availability, and the short time available for the data collection.

Search Terms for Data Collection

There were a few terminologies used to answer the fundamental question for the review. These key-words are as follows: synaptic function of neurons and memory formation, hippocampus and memory formation, synaptic plasticity and long-term potentiation, Alzheimer's disease and synaptic dysfunction, engram cells and memory

Inclusion and Exclusion Criteria

To do a systematic review, we have selected data following strict inclusion and exclusion criteria. We have included studies from the year 2013 to 2018. There is one study used in the introduction which is from 1998. However, we have not used the data from this study in our discussion section. For the core data collection, we have screened clinical trials along with rodent experiments.

Ethical Issues

No ethical issues were noted in the articles reviewed.

Quality Assessment

This is a peer-reviewed paper and was assessed by the AMSTAR checklist. The pool of searched and collected data was obtained from various journals whose impact factor lies from 0.12 to 40.137. We have also analyzed the publication type of the journals: academic journal publication; review articles; laboratory research; experimental research; research support, non-US gov't; research support, NIH extramural; research support, US gov't, Non-PHS from January 2013 to June 2018.

Results

Study Selection

**Figure 1:**
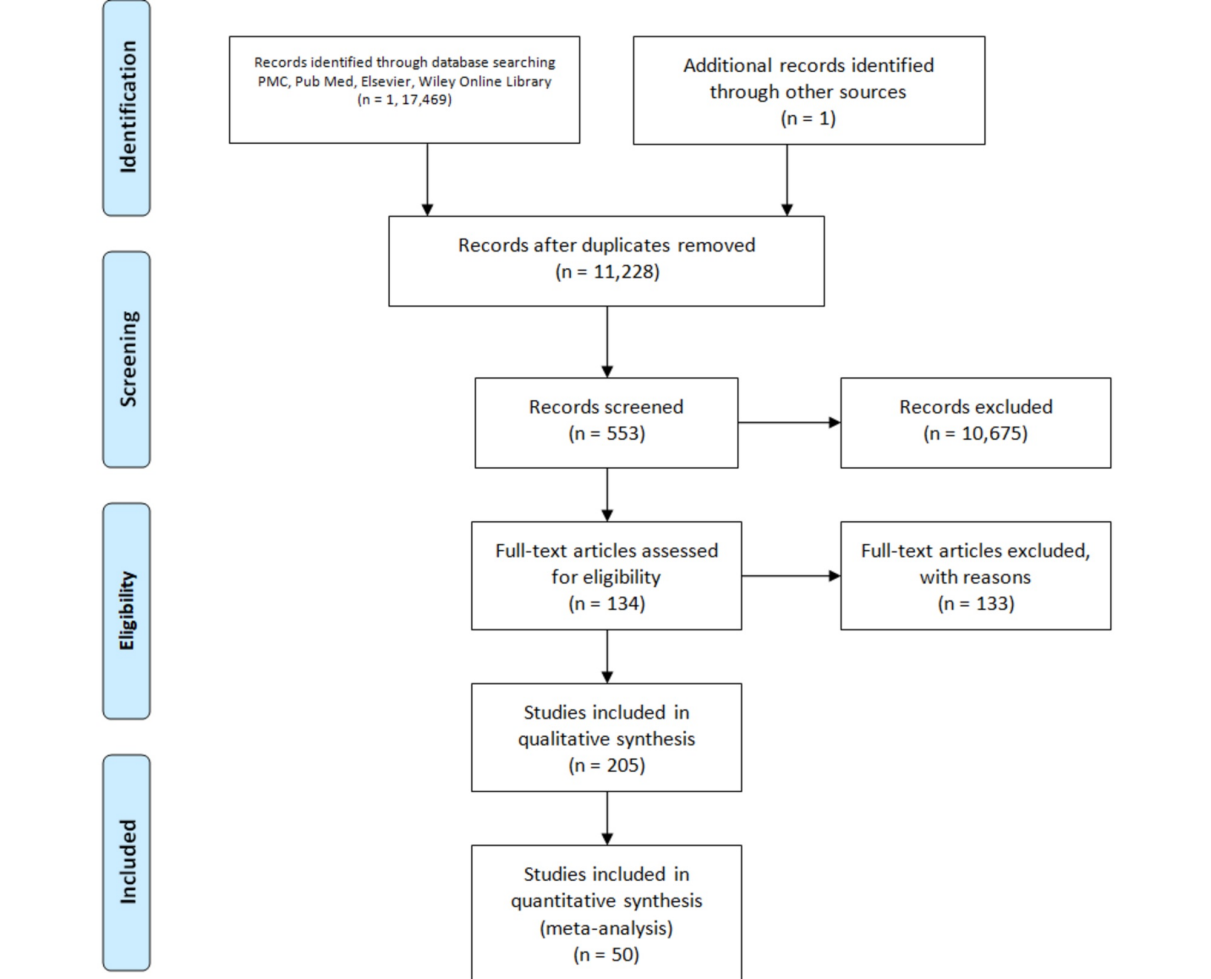
Prisma flow diagram

Initially, a total of 117,469 studies were identified from an electronic database search. After applying both inclusion and exclusion criteria, 44,911 were retained for the analysis. Reviewing such a large database yielded many duplicates, which were removed as precisely as humanly possible by comparing the title, authors, and metadata. In total, 11,228 studies remained after deduplication. Among the 11,228 studies, 553 studies were screened based on the title and abstract, out of which 205 potentially relevant studies were identified. Of these, 134 publications were ruled out after their full-text was found to be unavailable. A total of 50 studies were selected and included in our analysis. Two Japanese and one Spanish article were also included as translations were found in PubMed. The PRISMA flow chart for the process is shown below (Figure 1).

Discussion

Physiological/Structural and Functional Connectivity and Memory Formation and Synaptic Plasticity - Learning and Memory

Molecular, cellular, and chemical basis of memory: Changes in the neuronal circuit of the hippocampus leads to memory formation. By integrating neuronal function, and improving the strength of their connections, synaptoplasticity is improved and consolidated [11]. Episodic memories relay in the hippocampus and the associated structures. It is believed that episodic memory and spatial coding share the same circuitry and algorithms. There have been recent advances in understanding the cooperation and specialization of bilateral hippocampi, the role of synaptic plasticity in gamma phase-locking of spikes and place cell formation, a prefrontal-thalamo-hippocampal circuit for goal-directed spatial navigation [7]. For investigating the neural concepts behind memory, some studies focus on the hippocampus and medial temporal lobe (MTL) structures. To understand how memory signals from the hippocampus affect motor action, researchers proposed a new approach which includes looking at behavior and memory. Researchers show that it depends on the hippocampus, and how eye movements modify hippocampal neural signals [11].

Hebbian plasticity, which accounts for long-term potentiation and long-term depression, was once considered as being critical for local circuit representation regarding memory formation and behavioral change and stabilization in the mammalian central nervous system. On the other hand, some studies favor the concept of non-Hebbian, homeostatic forms of plasticity, which is synaptic scaling and command over intrinsic membrane characteristics for neuronal circuit development and stabilization. Use-dependent neurodevelopment was found to be affiliated with cell-wide modifications in postsynaptic receptor density. Recent cellular neurophysiology studies have revealed the crucial roles of transmembrane signal transduction, N-methyl D-asparted (NMDA) regulation, and regulation of neural membrane biophysical properties in behavior-modifying circuits. Regulation of gene transcription has been shown to bridge experiences and behavioral changes. Both, active DNA (de)methylation and regulation of chromatin structures, are considered to be critical governors of gene transcription for learning. One of the turning points for studies on memory formation and the learning process is the discovery of the protein synthesis dependence theory. Interference in these process leads to intellectual disabilities and memory disorders which will be discussed later in this article [10].

The process of memory formation involves the transcription to circuit modification, which in turn is caused by the assembly of particular neurons changing excitability and synaptic activation, followed by selective strengthening of pre-existing synapses, the formation of new connections, and the elimination of out-competed synapses [12]. The process of synaptoplasticity requires several proteins and also steroids. Proteins recruited in the synapses are required to be expressed by highly regulated DNA methylation histone tails post-translational changes. Neurosteroids might play a useful role in neurodegenerative therapies [11]. The direction for future research on the functional role of anti-inflammatory cytokines in the synaptic plasticity and cognitive functions of the human brain is wide open because preventive and therapeutic measures for neuropathologies require an understanding of the functional role of cytokines in the cellular mechanisms of storage and memory formation [13]. Kato et al's model on dopamine (DA) and reward prediction error (RPE) provides potential explanations for key experimental findings that suggest DA's roles in motivation: (i) deceleration of behavior by preventing post-training DA signalling, (ii) observations point that a DA blockade can cause severe impairment in obtaining rewards achieved with effortful actions as compared to less effortful, easily obtainable rewards, and (iii) between the reward amount, the level of motivation reflected in the pace of behavior, and the average level of DA. Moreover, it is believed that biological systems for value-learning might be in a state of equilibrium where learning and forgetting are balanced [14]. Short-term synaptic depression plays an important role in the processing of memory, which in turn can be quickly activated in response to a related stimulus [15]. A few models have been proposed, which contribute to the knowledge about synaptic proteins and their functions. Shreds of evidence suggest that there is a link between the accumulation of synaptic proteins and neurodevelopmental, neuropsychiatric, and neurodegenerative diseases. Synaptic proteins, mainly from excitatory synapses, might be related to several synaptopathies [16].

Studies on the origins of synapses and neurons have not produced clear results. For the sake of simplicity, researchers have studied the closest unicellular relatives of metazoans, which provide detailed information of when and how the first pre- and postsynaptic signaling mechanisms evolved [17]. Evidence suggests that postsynaptic regulations of α-amino-3-hydroxy-5-methyl-4-isoxazolepropionic acid-type glutamate receptors (AMPA-Rs) are critical for synaptic plasticity. To obtain long-lasting synaptic plasticity, local synapses need to be connected with activity-dependent, newly produced plasticity-related molecules in neuronal cell bodies. According to recent studies, the activity-dependent, memory-related protein, Arc, is a part of the synapse-specific modulation of AMPA-Rs. This Arc-based mechanism, together with other molecular mechanisms, might help maintain the contrast of synaptic strength between strong and weak synapses and help the genesis of long-term memory [18]. Engrams are believed to play a part in the cellular mechanism of memory storage. Although engram cells are distributed throughout the brain, the baso-lateral amygdala (BLA) is considered as the engram hub [19]. Research suggests that synaptic correlation for memory formation depends upon a stronger structural and functional affinity between engram cells across two directly connected brain regions [20]. Understanding these cellular and network mechanisms is necessary to determine the roles of emotional memory formation and storage in the healthy and pathological brain [19]. Reelin, a large extracellular matrix protein, performs a fascinating role in neurogenesis, neuronal migration in the cerebral cortex and the hippocampus, polarization, and synaptic plasticity. Reelin a core substance for the signaling cascade for learning and memory. However, the specific mode of action of the reelin cascade at the cellular and molecular levels remains unclear [21].

Among the several mechanisms, one of the major cellular mechanisms to lead the plasticity of neuronal networks for learning and memory is dynamic, and number of dendritic spines, as well as the synaptic incorporation and removal of AMPA-type glutamate receptors (AMPAr). In synaptic development and plasticity, it is believed that a critical role is played by the actin cytoskeleton, the Rho subfamily of GTP-binding proteins. Dedicated guanine nucleotide exchange factors (GEFs) and GTPase-activating proteins (GAPs) regulate shuttle between the active GTP-bound form and the inactive GDP-bound form. However, the function of GEFs and GAPs in the brain has not been yet clarified [1]. Actin cytoskeleton, located in dendritic spines, plays a vital role in maintaining synaptic plasticity at excitatory postsynaptic sites. Drebrin regulates actin cytoskeleton. Between the two F-actin pools of dendritic spines, accumulation of drebrin-decorated stable F-actin (DF- actin) is inversely regulated by the intracellular Ca2+ concentration. In LTP, the Ca2+ increase via N-methyl-D-aspartate (NMDA) receptors soon returns to the basal level, and AMPAR expression at the postsynaptic membrane is increased. The Ca2+ recovery and AMPAR increase are harmonized and induce the re-accumulation of DF-actin and change the dendritic spines from the excited state to a steady state during LTP maintenance. AMPAR endocytosis is facilitated due to the inhibition of re-accumulation of DF-actin caused by a prolonged increase in intracellular Ca2+ during LTD. Because of the positive feedback loop during AMPAR decrease and drebrin re-accumulation inhibition, the dendritic spines are found to be unstable during LTD maintenance [22]. The research on the effect of sirtuin 2 (SIRT2) on synaptic plasticity and cognitive function has demonstrated that SIRT2 acts as an AMPAR deacetylation and causes protein accumulation. Thus, the SIRT2 expression is accompanied by impaired learning and memory. AMPAR acetylation is associated with fast excitatory synaptic transmission, and synaptic plasticity, learning, and memory [23].

Brain-derived neurotrophic factor (BDNF), which is a protein, is involved in the regulation of long-term potentiation, long-term depression, and memory formation. BDNF propeptide is endogenously secreted from neuronal cells and is believed to multiply the posttranslational mechanism, which in turn enhances the biological action of BDNF [24]. BDNF and its high-affinity receptor, tropomyosin receptor kinase B (TrkB), is critical in memory destruction. Studies have already shown that c-Jun N-terminal kinase (JNK) interacted protein 3 (JIP3) mediates the anterograde axonal transport of TrkB via the direct connectivity of its coiled-coil area 1 (CC1) with TrkB. With the help of fluorescent CC1 and enhanced green fluorescent protein (EGFP) fused protein, CC1-EGFP, it is found that CC1-EGFP could notably disrupt TrkB anterograde axonal transport, and its accumulation at the presynaptic site [25]. Research has suggested three main features: “the facilitation of plasticity by BDNF, the postsynaptic of which activates Cell division control protein 42 (Cdc42) and Ras-related C3 botulinum toxin substrate 1 (Rac1), but not Ras homolog gene family, member A (RhoA); heterosynaptic facilitation of structural long-term potentiation (sLTP), which is carried by diffuse Rac1 and RhoA activity; and input specificity, which is provided by spine-restricted Cdc42 activity” which established the concept of biochemical computation in dendrites, secured specificity, and gave the system plasticity [26].

Research over the years has indicated that the ubiquitin-proteasome pathway (UPP) is critical for neurotransmission and synaptic plasticity via regulating presynaptic and postsynaptic proteins. Apart from UPP, lysosomes and autophagy also play a role in synaptic plasticity and memory using the same mechanism [27]. Integrins are an extracellular matrix that are present at high levels in synapses. The tetrapartite structure of the synapses is comprised of pre- and postsynaptic neurons, the extracellular matrix (ECM), and the glial processes. Thus, understanding the synaptic integrin function might lead us to information on synaptic plasticity and the memory mechanism [28]. Zn- dependent module tests the role of histone deacetylases (HDACs) in healthy neurons. This demonstrates the need for HDACs in normal olfactory memory retention. Moreover, electrophysiological experiments have suggested that learning deficits could be the behavioral consequence of HDCA6 mutants [29]. Less attention has been paid to extracellular Zn2+ for synaptic activity as compared to Ca2+. An increase in the basal level of Zn2+ is age-dependent, and it is linked with age-related cognitive function and dysfunction. Researchers suggest that extracellular Zn2+ influx might be linked with the weakened intracellular Zn2+ buffering in the aged dentate gyrus [4].

The neocortical microcircuits' mechanism, that encodes and replays sequences, elicits a particular behavior pattern to a certain stimulus. With the help of the Bayesian Confidence Propagation Neural Network (BCPNN) learning rule, the researchers found out that the formation of distributed memories, including increased periods of firing in the pools of excitatory neurons, together with asymmetrical associations between distinct network states, can be acquired through plasticity. It is believed that stimulus duration, level of background noise, the ratio of synaptic currents, and the strengths of short-term depression and adaptation determine learning and the speed of sequence replay [30].

Experience-dependent changes in synaptic efficacy and neuronal connectivity in the brain play a role in learning. Research by Chiu et al. contributes direct evidence for “physiological roles of the recycling endosome protein (GRASP1) in glutamatergic synapse performance, and animal conduct." Some studies show a probable role for GRASP1 in the pathophysiology of human cognitive disorders. This can be justified by some studies that show that the convergent disruptive effects on AMPAR recycling and glutamate uncaging might be due to two GRASP 1 point mutations in intellectually disabled patients [31]. Several mechanisms for homeostatic plasticity of excitatory synapses have been identified. Emerging studies direct a link between impaired homeostatic synaptic plasticity and neuropsychiatric and neurologic disorders. Hebbian forms of synaptic plasticity, such as long-term potentiation (LTP), inspire long-lasting modifications in synaptic strength, which can be sabotaging and lead to saturation. On the contrary, homeostatic plasticity operates to indemnify extensive activity changes, stabilizing neuronal firing within a dynamic physiological range [32]. Learning and memory, maladaptive behaviors - anxiety disorders, phobias, and post-traumatic stress disorder - have experience-dependent plasticity which involves both structural and functional alterations that result in adaptive behaviors. Experience-dependent plasticity is not similar to synaptic plasticity. Changes in the number, distribution, or activity of various ion channels situated throughout the neuron are called intrinsic plasticity. The formation of memories and, more interestingly, the participation of neurons in a memory trace can be understood by intrinsic excitability theories. Most importantly, with the help of modified intrinsic excitability, learning abilities can be regulated. This, in turn, not only prevents or treats functional cognitive disorders, but it also improves normal cognitive function in an ageing population [33].

Genetic basis behind memory formation: Recent pieces of evidence have suggested that apart from molecular, cellular, and chemical mechanisms, genetics play a huge role in memory synapse formation and dysfunction, leading to neurodegenerative diseases.

Long-term potentiation is a possible mechanism for learning and memory formation. Studies show that long-term potentiation causes a persistent strengthening of synapses following a high level of stimulation. This strengthening requires a series of complex molecular processes, and the synchronized reconditioning of presynaptic and postsynaptic neurons. Although the research on the transcriptional machinery and molecular processes underlying LTP has been going on for decades, yet it stays incomplete. For a greater understanding of molecular mechanisms related to learning and memory formation, more studies on recognizing all the proteins and non-coding RNA transcripts expressed during LTP might be helpful [34]. Long-term and short-term memories are primarily categorised based on the period of their memory withholding capacity. At a molecular level, long-term memory (LTM) differs from short-term memory (STM) by its need for new gene expression. Besides transcription (nuclear gene expression), the translation of mRNAs is also essential for LTM formation. Currently, the major problem of interest in molecular, cell biological, neurobiological and clinical perspectives is the mRNA mechanism and function for temporal and spatial control required for LTM. The preliminary evidence for the notion is the Cytoplasmic Polyadenylation Element Binding (CPEB) protein, which allows the perseverance of organized memories by altering in a prion-like manner from a soluble monomeric state to a self-maintaining and consistent polymeric translationally active state necessary for perpetuating persistent synaptic plasticity. Emerging evidence suggests that such RNA regulatory proteins are constituents of mRNP (RiboNucleoProtein) granules. In proteins, prion-like domains, being solely disorganized, could engage in feeble short-termed interactions that permit the gathering of RNP granules, a source of suppressed mRNAs whose translation is essential for LTM [35].

Current studies show that the inhibition of adult neurogenesis attenuates the emergence of the hippocampus-relying on memory. Researches indicate that the conditional and targeted knock-out of ERK5 MAP kinase in adult neurogenic regions of the mouse brain impairs adult neurogenesis in the hippocampus and hampers different forms of hippocampus-dependent memory. It is reported that adult neurogenesis in the dentate gyrus is the end product of selective and targeted activation of ERK5 by magnifying cell survival, neuronal differentiation, and dendritic complexity. Conditional ERK5 activation also boosts the production of demanding forms of spatial learning and memory and increases hippocampus-dependent long-term memory. To extend neurogenesis for upgrading the stamina of hippocampus-dependent memory, improvement in the signal transduction of a single signaling pathway within adult neuronal stem/progenitor cells is enough. Moreover, activation of ERK5 may direct to an advanced therapeutic target to enhance long-term memory [36]. Epitranscriptomic mechanisms are considered as an emerging field which includes neural plasticity, learning, and memory. Studies have shown that the effect of RNA modulations on activity-induced RNA structure states, RNA editing, and RNA localization and qualitative state differs in RNA elevate the functional variety and information-carrying potentiality of RNA molecules [37]. Researchers have proved that human and mouse synaptic activity-induced transcriptional programs need positively regulated synapse-to-nucleus signaling. To understand the evolution of human cognitive abilities, various species-specific models have been proposed. The mouse genome is lacking in non-coding RNA genes BRE-AS1 and long intergenic non-protein coding RNA 473 (LINC00473) and protein-coding gene zinc finger protein (ZNF331), which may indicate that lineage-specific gain of genes and DNA regulatory elements influence the synaptic activity-regulated gene program during evolution [38].

What if Normal Synaptic Function is not Maintained?

Overview of neurodegenerative disorders: Embryonic and fetal periods have the highest developmental neuroplasticity. However, the gradual depletion in plasticity is never complete, and the ability to adapt the brain circuits in response to naïve learning is called adaptive neuroplasticity; brain injuries are called reactive neuroplasticity, rests throughout the individual's whole lifespan. The brain’s lack of adaptive or reactive plasticity to upgrade skewed or impaired circuits by genetic or environmental anomalies might lead to neurodevelopmental disorders [39].

Reelin plays a fascinating role in neurogenesis; neuronal migration in the cerebral cortex and the hippocampus; and the polarization and synaptic plasticity with its implications in learning and memory. However, the specific mode of action of the reelin signaling cascade at the cellular and molecular levels remains unclear. Although some shreds of evidence have suggested that such core pathways are apolipoprotein E receptor 2 (ApoER2), low-density lipoprotein receptor-related protein 8 (LRP8), and very-low-density lipoprotein receptor, followed by proto-oncogens like Src/Fyn-mediated phosphorylation of the adaptor protein Dab1 (Disabled-1). Phosphorylated Dab1 (pDab1) is reviewed as a core in the signaling cascade, from which several other downstream pathways divide, complementing the different roles of reelin. Many of these pathways influence the dynamics of the actin and microtubular cytoskeleton and membrane trafficking through the modulation of the activity of small GTPases, including the Rho, and Rap families and molecules took part in cell polarity [21].

Experience-dependent changes in synaptic efficacy and neuronal connectivity in the brain play a role in learning. Some studies show a probable role for GRASP1 in the pathophysiology of human cognitive disorders. This can be justified by some studies which show that the convergent disruptive effects on AMPAR recycling and glutamate uncaging might be due to two GRASP1 point mutations in intellectually disabled patients [32]. The formation, organization, and plasticity of excitatory synapses are controlled by Prosap/Shank scaffolding proteins. Most of the Shank is found at the postsynaptic membrane of glutamatergic neuromuscular junctions and dominates multiple criterions synapse biology relying on the dose. Studies have shown that Shank regulates a non-canonical Wnt signaling pathway in the postsynaptic cell by regulating the manifestation of Wnt receptor Fz2 [40]. A few models have been proposed to contribute to the knowledge about synaptic proteins, and their functions. Evidence has suggested that there is a link between the accumulation of synaptic proteins and neurodevelopmental, neuropsychiatric, and neurodegenerative diseases. Synaptic proteins, mainly from excitatory synapses, might be related to several synaptopathies [16].

Role of synaptic function defect in Alzheimer's disease (AD): Alzheimer's disease (AD) is one of the most common neurodegenerative disorders identified by a continuous loss of cognition, the existence of two hallmark lesions, senile plaques (SP), and neurofibrillary tangles (NFT), which result from the build-up and deposition of β-amyloid peptide (Aβ) and the aggregation of hyperphosphorylated tau protein, respectively. The new paradigm for AD etiology suggests there might be involvement of soluble oligomeric forms of Aβ as a root cause for cognitive deficits by particularly aiming synapses and damaging synaptic signaling pathways [8]. Recent studies also show that before the formation of amyloid plaques and neuronal cell loss, reduction in synaptic transmission, and loss of dendritic spines take place in AD. Postmortem human brains show a drebrin loss in postsynaptic sites before any presynaptic changes under immunohistochemistry. Studies show dysregulation of glutamate receptor trafficking and the p21-activated kinase/LIM kinase pathway present in AD brains. Aβ-derived diffusible ligands (ADDLs), which are soluble Aβ oligomer, attach to the postsynaptic site and produce the unusual morphology and density of dendritic spines. This observation suggests that at the primitive stage of the AD, before the density and morphology of dendritic spines changes, drebrin loss in dendritic spines happens. Thus, before the development of dementia in AD, quantitation of drebrin might provide a useful diagnostic tool [41]. The changes in dendritic spines are directed by modifications of F-actin dynamics under the command of Rho GTPases or by synaptic trafficking and insertion of glutamate receptors [9].

Structural involvement in AD manifests in the medial temporal lobe where neurofibrillary tangles accumulate and also in the loss of the neurons in the entorhinal cortex. With the help of serotonergic receptor modulating agents, recent preclinical studies on APP/PS1 (familial Alzheimer's disease) have demonstrated clear neuroprotective effects. We need to reassess the involvement of midbrain raphe in AD-induced dementia in an early stage. Indeed, a theory of serotonergic modulation of direct memory formation by explicit amplification of synaptic strength could change the view of the role of these nuclei in AD and lead to more potent treatments [42]. Neuropeptides manage hippocampal synaptic plasticity, the impairment of which might play a part in causing the cognitive deficits seen in Alzheimer's disease (AD). How the pro-opiomelanocortin/melanocortin 4 receptor (POMC/MC4R) system works in the hippocampus, and its part in a synaptic defect in the AD, are widely unknown, although POMC-derived neuropeptides and MC4R are compromised in hippocampus-hinging synaptic plasticity. However, it is believed that stimulation extricates amyloid-β-induced synaptic dysfunction via a Gs/cyclic AMP (cAMP)/PKA/cAMP-response element binding protein (CREB)-dependent mechanism. Hence, impairment of this hippocampal POMC/MC4R circuit might be the cause of the synaptic dysfunction seen in AD, disclosing an effective therapeutic target for the disease [43]. One of the essential organelles for the synaptic function is mitochondria. The special role of mitochondria in neurons is buffering C2+ and thus serving as local energy resources by providing ATP (adenosine triphosphate) to constant neurotransmitter delivery. One of the earliest and most prominent features in AD is mitochondrial abnormalities. The reason for mitochondrial dysfunction is an accumulation of amyloid-β (Aβ) and tau. We can develop a promising treatment strategy for AD by focusing on the importance of mitochondria in supporting synaptic function [44].

A genetic risk factor corresponding to AD is that the sortilin-related receptor with LDLR class A repeats (SORLA, SORL1, or LR11). Studies demonstrate that SORLA collaborates with the EphA4 receptor tyrosine kinase and diminishes ephrinA1 ligand-induced EphA4 clustering and activation to restrict downstream results of EphA4 signaling in neurons [45]. Mutation in presenilins in familial AD (FAD) is connected directly with synaptic Ca2+ signaling deregulation, probably by influencing the endoplasmic reticulum (ER) Ca2+ leak function of presenilins. Extravagant ER Ca2+ release via type 2 ryanodine receptors (RyanR2) is seen in AD spines due to the rise in and function of RyanR2. Store-operated Ca2+ entry (nSOC) pathway is hampered in AD spines because of the down regulation of the STIM2 protein. In the end, synapses are weakened and removed in brains by the LTD mechanism, leading to memory loss. Attacking synaptic calcium signaling pathways gives a chance for improvement of AD therapeutic agents [46].

Impairment in the cognitive and synaptic function of animal and cellular models of tauopathies promotes the theory of caspase-2 cleavage of tau at aspartate 314 (Asp314). In AD patients, the end product, Δtau314, repels fibrillation and is present at higher levels. Studies on the prevention of memory deficits and neurodegeneration suggest that by expressing the tau mutants which are resistant to caspase-2 cleavage prevents tau from invading spines, displacing glutamate receptors and reducing synaptic function in cultured neurons. Thus, preventing tau from infiltrating dendritic spines might provide scope for re-establishing synaptic function and treatment of AD [47]. Plenty of evidence suggests that the accumulation of toxins like soluble amyloid-β (Aβ) and hyperphosphorylated tau cause dysfunction of synaptic plasticity and the simultaneous release of irregular neurotransmitters (NT) at synapses proceed to cognitive decline as seen in Alzheimer’s disease (AD). Further, a disparity between excitatory and inhibitory neurotransmission systems inspired by aberrant redox signaling and changing mitochondrial integrity is also responsible for such abnormalities. Faulty NT release at the synaptic junction causes several pernicious effects related to the defective activity of synaptic proteins, transcription factors, Ca2+ homeostasis, and other molecules crucial for neuronal plasticity. These nasty effects further impair the normal homeostasis of neuronal cells, thus causing synaptic loss. Moreover, the precise mechanistic role played by defective NTs and neuromodulators (NMs), and impaired redox signaling in synaptic dysfunction stay in the dark, and their possible connection still needs to be investigated [48].

Progress in Pharmacological Aspect of Neurodegenerative Diseases Mainly AD Based on Normal Synaptic Function

There have been several advances in understanding neurodegenerative disorders and new treatments have been developed with an understanding of synaptic mechanisms. We have tried to cover some of these in this review article.

The direction for future research on the functional role of anti-inflammatory cytokines in synaptic plasticity and the cognitive functions of the human brain is wide open because preventive and therapeutic measures for neuropathologies require an understanding the functional role of cytokines in cellular mechanisms of storage and memory formation [13]. Changes in the neuronal circuit of the hippocampus lead to memory formation. By integrating neuronal function, and increasing the strength of their connections, synaptoplasticity is improved and consolidated. The process of synaptoplasticity requires several proteins and steroids. Neurosteroids might play a useful role in neurodegenerative therapies. Proteins expressed at synapses are highly regulated by DNA methylation, and histone tails posttranslational modifications [11]. Subunits of tubulin form cytoskeletal polymers of microtubules (MTs). Recent studies show that MTs are more active and polarize directly in the dendrites of neurons in the postsynaptic part of excitatory neurons in CNS. In degenerative diseases like AD, MT dynamics might be compromised. This gives us the prospect of attacking MT dynamic with the help of newer therapeutic agents [49]. Perineuronal nets (PNNs) are special extracellular matrix structures that loop around particular neurons in the CNS, during development, and control plasticity in the adult CNS. Studies show that they play a wide range of roles in diseases/disorders of the brain; participate in recovery from spinal cord injury; and are changed during ageing, learning, and memory, and after manifestation to drug abuse. Understanding the molecular underpinnings of how PNNs are altered in disease will provide awareness into new treatment modalities for these diseases. More studies on the molecular basis of involvement of PMN in normal physiology and disease might be helpful in the further development of disease-modifying agents [50]. Disruption in hippocampal synaptic plasticity by neuropeptides might lead to a cognitive deficit of AD. Pro-opiomelanocortin (POMC)-obtained neuropeptides, and melanocortin 4 receptor (MC4R) is involved in hippocampus-dependent synaptic plasticity. MC4R in the CA1 is activated by POMC neurons located in the cornu ammonis 3 (CA3). Amyloid-β-induced synaptic dysfunction is rescued by MC4R activation. The mechanism behind this action is Gs/cyclic AMP (cAMP)/PKA/cAMP-response element binding protein (CREB)-dependent. Hence, interference of this hippocampal POMC/MC4R circuit might lead to the synaptic dysfunction seen in AD, suggesting a potential therapeutic target for the disease [44].

Mutation in presenilins in the AD (FAD) is connected directly with synaptic Ca2+ signaling deregulation, probably by influencing the endoplasmic reticulum (ER) Ca2+ leak function of presenilins. Extravagant ER Ca2+ release via type 2 ryanodine receptors (RyanR2) is seen in AD spines due to the rise in expression and function of RyanR2. The store-operated Ca2+ entry (nSOC) pathway is hampered in AD spines by down regulation of STIM2 protein. In the end, synapses are removed in AD brains by the LTD mechanism, leading to memory loss. Attacking synaptic calcium signaling pathways gives a chance for improvement of AD therapeutic agents [47].

The distinct role of intrinsic excitability can give insight into how memories are made and, more interestingly, how neurons that engage in a memory trace are chosen. For prevention or provision of treatment in not only in patients with clinical disorders, but also in ageing population, the modulation of intrinsic excitability which in turn regulates learning ability is very important [34]. The possibility for the pharmacological aspect of neurodegenerative diseases is humongous. We suggest doing more research on this topic to make a better cure for nearly incurable diseases like Alzheimer's in the future.

Limitations

We might have missed some important studies due to the screening of only the abstracts. Moreover, because of manual search and comparison, we might have amiss in duplication counting.

## Conclusions

Humankind is making a mark in every field in the 21st century. Now it is time to focus on a phenomenon which is considered to be impossible to cure. In this review, we have tried to approach the question "connection between synapses and memory, role of synapse modification in neurodegenerative diseases.” The whole concept of a memory synapse rests on the potentiality of the animal to make changes in its model of the world through new information. Thus, there must be an ability to delete these structures when their description of the world is no longer correct. Last but not least, we have also discussed the possible primary etiology of neurodegenerative diseases, mainly Alzheimer's disease, concerning synaptic dysfunction. With the help of high tech machines and the better understanding of the disease etiology, we can not only identify the missing pieces of the concept of synaptic functions, but also we might be able to cure or even prevent severe neurodegenerative diseases like AD.
